# Photokinetics of Bimolecular Reactions: Analytically Solvable Rate Laws

**DOI:** 10.3390/molecules31010084

**Published:** 2025-12-24

**Authors:** Mounir Maafi

**Affiliations:** Leicester School of Pharmacy, De Montfort University, The Gateway, Leicester LE1 9BH, UK; mmaafi@dmu.ac.uk

**Keywords:** Φ-order kinetics, photokinetics, bimolecular reaction, quantum yield, analytical integration

## Abstract

Light-induced bimolecular reactions occur in many naturally and artificially (laboratory or industrial) designed processes. The quantification of these reactions is generally performed by kinetics. In particular, the kinetic data of bimolecular photoreactions are often treated by second-order kinetic models. If this situation is effectively ubiquitous in practice, it remains that the underlying hypothesis, assuming that photoreactions obey the same kinetics as thermal transformations, is not consistent with the physical photosystem considered. In fact, it has been proven that unimolecular (mono-reactant) photoreactions are effectively modelled by Φ-order kinetics. The latter model is formalised by a logarithmic function bearing an exponential in its argument. Hence, Φ-order kinetics is mathematically different from the thermal reaction models. In the case of the bimolecular photoreactions that are described by different rate laws than those used for the thermal reactions, i.e., involving both radiation intensity and light absorption, there have been no reported solutions in the literature that were based on analytical integration. So much so, no kinetic order has ever been assigned to any bimolecular photoreaction. In the current situation, it is perhaps sensible to proceed, in a first step, by defining among the bimolecular photoreactions those whose rate laws can be solved analytically and establish the corresponding solutions by closed-form integration. Following such a strategy, the present paper unravels the first model equations for the kinetics of bimolecular photoreactions. The findings are part of an effort to standardise photokinetics along the same principles used in thermal kinetics.

## 1. Introduction

Bimolecular photoreactions have been known since the very beginnings of photochemistry. A succinct historical review [1] proclaimed that the first known organic [2 + 2] cycloaddition reaction was reported in the late nineteenth century [2,3], whereas the first well-documented reaction between a carbonyl compound and an alkene was published in 1909, which latter led to the establishment of the Paternò–Büchi mechanism [4,5]. By 1975, the number of photoreactions suitable for organic synthesis had risen to 8000, and the number is still growing [6,7].

Organic photochemistry has an appealing potential in sustainable chemistry [8,9,10], and many photoreactions are part of established industrial processes [11,12]. Light-initiated reactions have proposed new biologically active molecules for various applications in pharmacy, medicine, and fine chemistry [13,14,15,16,17], and play a pivotal role for other important technological fields such as visible light photocatalysis [18], atmospheric photo-oxidation [19], solid state photoreactions for medicinal dosage forms [20], single crystals [21], photodimerisation [22,23], photopolymerisation [24,25], and continuous-flow photochemistry [26,27].

The quantification of bimolecular photoreactions may be achieved by two means: either by kinetics or by direct measurement of one or more components of the reactive medium at a given time. The latter approach is often performed by the measurement of the concentration or a percentage at a given time span [28,29,30,31]. For the former method, the kinetics of such photoreactions are generally performed using the same models employed for thermal reactions. The kinetic data were generally treated by second-order, pseudo-first-order, or first-order equations [20,31,32,33,34,35,36,37,38]. It is interesting, in this context, to consider the lack of explanation or justification relative to the change of reaction order occurring for bimolecular photoreactions [39], or the reported disagreement on whether the decay of charge carriers in photocatalysts obeys first- or second-order kinetics [40,41]. It is also noticeable that this kind of question is still standing today. In addition, the pertinence of using the thermal kinetic models for photoreaction can also be challenged based on the fact that the mathematical formulation of the thermal kinetic models does not involve the radiation intensity [42,43,44]. In some instances, however, the rate law of a bimolecular photoreaction included the incident light intensity [24,45]. But such formulations did not analytically derive integrated rate laws for bimolecular photoreactions (see Section 2.2).

The quantification of bimolecular photoreactions is also achieved by determining the quantum yield, which is typically calculated by using either the differential or integral quantum yield classical formulae [46]. The differential quantum yield obtained through a pseudo-first order treatment of Paternò–Büchi photoreactions was reported to vary with the initial concentration of the (second) most concentrated reactant [34].

The variety of the kinetic methodologies and the forms assigned to the rate law may raise two important concerns: On one hand, that a consensus on a single formulation of the rate law for bimolecular photoreactions has not yet been reached in the community. And on the other hand, the explicit expression of the integrated rate law, in general or for specific cases, is lacking in the literature.

The present paper tackles this problematic, isolates the cases for which the rate law can be integrated in a closed form, and gives the formulae of the integrated rate laws for those cases. The proposed formulae of integrated rate laws represent the first examples for bimolecular photoreactions whose rates include the incident light, and hence, will contribute to standardising photokinetics along the general rules and concepts of kinetics.

## 2. Results and Discussion

### 2.1. Different Bimolecular Photoreactions

**Scheme 1:**
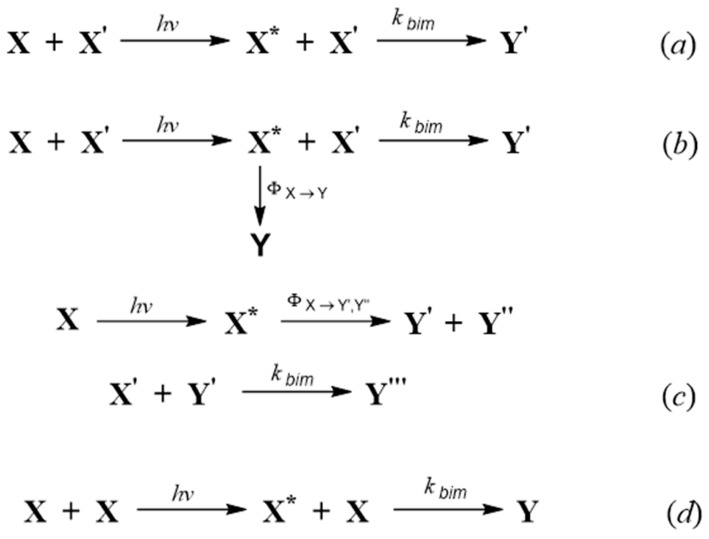
Most prominent bimolecular reactions (**a**–**d**).

The basic bimolecular photoreaction implies the reaction of a reactant molecule (X) in its excited state ($X^{*}$), after absorption of a photon of light, with another molecule (a second reactant, e.g., $X^{\prime }$) in its ground-state to yield a product ($Y^{\prime }$), as shown in Scheme 1a. There are, however, a few other cases that can be considered as bimolecular reactions, of which the most recurrent ones are depicted in Scheme 1.

In one case, the reactant in the excited state can also simultaneously be depleted by a concurrent reaction to the ongoing bimolecular process, to form a different product, Y (Scheme 1b). Another reaction situation involves the direct photoreaction of the excited reactant, which might photoconvert, in a first step, into one or more products in their ground states (e.g., $Y^{\prime }$ and $Y^{\prime \prime }$), which can, in a second step, further thermally react with the second reactant ($X^{\prime })$ to generate a new product (e.g., $Y^{\prime \prime \prime }$, Scheme 1c). The excited species ($X^{*}$) can also react with a homologue ground-state molecule (X), to produce a new compound (e.g., a dimer, Scheme 1d).

In Scheme 1, the impinging light beam on the sample, indicated by hv, activates the photoreactive reactant, X, but can also concomitantly be absorbed by the other species present in the medium. A direct photo-transformation is labelled by a photochemical quantum yield, such as $\Phi_{X\;\to \;Y}^{\lambda_{irr}}$ or $\Phi_{X\;\to \;Y^{\prime },Y^{\prime \prime }}^{\lambda_{irr}}$. The quantum yields are necessary referred to the wavelength of the incident light ($\lambda_{irr}$) that is absorbed by the photoactive reactant, X, and implicitly includes the loss processes occurring along the considered reaction path, e.g., thermal relaxation of the excited-state species to the mother molecule ($X^{*}\;\to \;X$), and emission ($X^{*}\;\to \;X+hv\prime$). The subsequent thermal reaction is characterised by the bimolecular rate constant $k_{bim}$. The photochemical quantum yield is specific to the light-absorbing species at the start of a photochemical reaction step and its conversion into a product under exposure to a strictly monochromatic light. Whereas the quantum efficiency has a much broader meaning, measuring, for instance, the ratio of the product generated versus the incident photons [44,46].

Kinetically, the bimolecular reactions shown in Scheme 1 are different because they are described by different rate laws. Mathematically, such differences in the rate laws should, in the general case, lead to different integrated rate laws [44].

### 2.2. The Kinetic Treatment in the Literature

The kinetic approaches proposed for the analysis of photoreactions can be divided into two options. One considers the steady state, and the other deals with the dynamic reactive system.

The first option, the steady-state approximation, considers that the concentration of the excited-state species remains relatively constant over time (assuming the occurrence of an equilibrium between the production and depletion of this excited-state molecule, e.g., $X^{*}$). In this perspective, the rate of change of the excited-state species concentration is set to zero since its formation and disappearance rates are supposed to be equal. This technique is generally used in photochemistry to define a simple equation for the quantum yield of the reaction at hand. Although this method has the advantage of being easy to implement and the equations delivered have a relatively low number of parameters, the steady-state method remains only useful when the species concerned are in an equilibrium state, some of the inaccessible parameters of the equation are possibly approximated or made negligible, and the dynamic processes are not considered [41].

The dynamic kinetic analysis, as the second option, is achieved by considering the rate law of the reaction and the continuous variation of the concentrations over time.

The ubiquitous kinetic model applied to the treatment of bimolecular photoreactions’ data is that of the second-order thermal reaction ($X+X^{\prime }\;\overset{k_{∆}}{\to }\;Y$, with $k_{∆}$ being the bimolecular rate constant of the thermal reaction) [21,23,29,38,47]. In this case, the rate is proportional to both the temperature-dependent $k_{∆}$ and the initial concentrations of the reactants, as

(1) $$r_{X}^{T}\left(t\right)=\frac{dC_{X}^{T}\left(t\right)}{dt}=-k_{∆}\;C_{X}^{T}\left(t\right)\;C_{X^{\prime }}^{T}\left(t\right)\;$$

When the concentration of one reactant (e.g., $X^{\prime }$) is set to a much higher value compared to that of the other reactant (X), the concentration of the former can be deemed constant during the reaction progress, and hence, can be incorporated into the reaction rate constant, $k_{∆}^{\prime }=k_{∆}\;C_{X^{\prime }}^{T}\left(t\right)$. Under this condition, the rate will be proportional to only one variable, i.e., $C_{X}^{T}\left(t\right)$, and the overall reaction will now obey first-order kinetics, hence dubbed the pseudo-first-order kinetics [28,31,45,48]. Reciprocal second-order kinetic models have also been reported [30,33,38,45,49,50,51] when the rate is proportional to the square of the concentration (or when the values of the reactants’ initial concentrations are chosen to be equal, $C_{X}^{T}\left(0\right)=C_{X^{\prime }}^{T}\left(0\right)$).

Even though such a kinetic description provides handy equations that are easy to use, it fails to provide a consistent account of the bimolecular photoreaction dynamics. Indeed, Equation (1) does not explicitly involve the radiation intensity, an important parameter for photoreactivity. This parameter might be supposed to be part of $k_{∆}$, but in such an assumption, the unit of the rate constant must change (which is not reported in the literature). Also, in its present formulation, Equation (1) does not differentiate between the type of the incident light used, whether it is mono- or polychromatic. Such an amalgamation is detrimental to a comprehensive description of the kinetics. Of the same level of significance, Equation (1) falls short on displaying how the absorbed light impacts the reaction (from the viewpoint of absorption by the species present in the medium). More importantly, Equation (1) considers that all the molecules of X, present in the medium at reaction time t, are potentially involved in the bimolecular reaction. If this is true for the thermal reaction, it cannot be validated for a photoreaction since only a fraction of $C_{X}\left(t\right)$ is effectively reactive, that is, the molecules of X being in their excited state at time t (i.e., $X^{*}$). Therefore, Equation (1) is not representative of the physical system embodied by bimolecular photoreactions. A similar conclusion has been reached by several authors for various photoreactions [41,42,52,53].

Other authors have adopted an approach where the characteristic rate law of bimolecular photoreactions explicitly involves the incident radiation intensity [21,23,29,38,45,47,54]. Most often, the time dependencies of the reaction species are ignored, due to difficulty in solving the complex rate laws, and instead the kinetic data treatment focuses only on the initial stages of the reaction [47,55].

Alternatively, taking the dynamics of the reaction into account requires starting from a rate law (explicitly involving the radiation absorption and intensity) and deriving the corresponding integrated rate law.

In general, the rate law is alleviated by, for instance, introducing an approximation using the power series expansion. The latter is usually reduced to its first-order form. By this procedure, the term bearing an absorbance in its exponent, $1-10^{-\varepsilon \;l\;C}$, becomes linear as, ε l C [20]. This operation transforms the non-linear rate law of the photoreaction into a linear differential equation. The closed-form integration of the new rate law is hence possible. It is important to keep in mind that such an approximation by using the first-order term of the power series expansion is only valid for very small values of the absorbance (ε l C≤0.01) [44]. A condition that is often overlooked in practice, represents experimental constraints and poses difficulty for reliable spectroscopic and analytical measurements.

As a matter of fact, no analytical integration of the original (non-linear) rate law of any bimolecular reaction has, thus far, been published in the literature.

### 2.3. The Differential Equation of the Rate Law

The rate law of the reactant of a unimolecular photoreaction (X→Y), in a slab-shaped and vigorously stirred reactor, and continuously exposed to a monochromatic/collimated light, has been well described in the literature [42,44,52,53,56,57,58]. It has been expressed as the product of the photochemical quantum yield and the fraction of the light absorbed by the reactant, as

(2) $$r_{X}^{\lambda irr,T}\left(t\right)=\frac{dC_{X}^{\lambda irr,T}\left(t\right)}{dt}=-\Phi_{X\;\to \;Y}^{\lambda_{irr}}\;P_{a_{X}}^{\lambda_{irr}}\left(t\right)\;$$

The basic light-initiated reaction that involves two reactants ($X+X^{\prime }\to Y\prime$) can be viewed as unfolding in two stages, first, the formation of the photoexcited state of one of the reactants by absorption of the incident light ($X\to X^{*}$), followed in a second step (Scheme 1a), where a thermal bimolecular reaction takes place between the latter species and the second reactant ($X^{\prime }$).

The rate of the reaction of species X must involve a term corresponding to the light absorption by X. This can be expressed by the amount of photons absorbed by the reactant, $P_{a_{X}}^{\lambda_{irr}}\left(t\right)$, which itself corresponds to the number of molecules of X in the excited-state (i.e., $X^{*}$, which is the only fraction of X that is capable of reacting with X′ at a given reaction time). In this sense, the first step of the bimolecular reaction (considered in the same experimental conditions listed above) is equivalent to the template of the unimolecular photoreaction (Equation (2), but without reaction, i.e., omitting the quantum yield). It is, however, useful to observe that only a fraction of the excited molecules ($X^{*}$) will effectively react (the rest will follow other paths that do not lead to a reaction). The intensity of the initial radiation is also explicit in Equation (2) as part of the $P_{a_{X}}^{\lambda_{irr}}\left(t\right)$ formulation (*vide infra* Equation (4)).

In the second stage of the reaction process, the excited molecules ($X^{*}$) thermally react with the second, ground-state reactant (X′) to eventually produce Y′ (with this reaction-step being characterised by the thermal bimolecular rate constant, $k_{bim}$).

Light has an irradiation wavelength of $\lambda_{irr}\;$(in nm), and the irradiated volume and area of the reactor ($V_{irr}$ in ${dm}^{3}$ and $S_{irr}$ in ${cm}^{2}$) are defined by the experimentalist. 

The overall rate equation for this bimolecular photoreaction (Equation (3)) takes the general formulation adopted for second-order thermal reactions (Equation (1)), but with the rate being dependent on both the incident radiation intensity, $P_{0}^{\lambda_{irr}}$ (which dimension is $einstein\;{dm}^{-3}s^{-1}$), the species absorptivities, ε (in $mol\;{dm}^{-3}\;{cm}^{-1}$), $\lambda_{irr}$, and the constant medium temperature (T), as

(3) $$r_{X}^{\lambda_{irr},T}\left(t\right)=\frac{dC_{X}^{\lambda_{irr},T}\left(t\right)}{dt}=-\frac{dC_{Y^{\prime }}^{\lambda_{irr},T}\left(t\right)}{dt}=-r_{Y^{\prime }}^{\lambda_{irr},T}\left(t\right)=-k_{bim}\;P_{a_{X}}^{\lambda_{irr}}\left(t\right)\;C_{X^{\prime }}^{\lambda_{irr},T}\left(t\right)\;$$

The fraction ($P_{a_{X}}^{\lambda_{irr}}\left(t\right)$) of the incident monochromatic light that is absorbed by the reactant X, which is expressed in the same units of $P_{0}^{\lambda_{irr}}$, is given by

(4) $$P_{a_{X}}^{\lambda_{irr}}\left(t\right)=\frac{A_{X}^{\lambda_{irr},T}\left(t\right)}{A_{tot}^{\lambda_{irr}T}\left(t\right)}\;P_{0}^{\lambda_{irr}}\;\left(1-10^{-A_{tot}^{\lambda_{irr},T}\left(t\right)}\right)=A_{X}^{\lambda_{irr},T}\left(t\right)\;P_{0}^{\lambda_{irr}}\;\frac{1-10^{-A_{tot}^{\lambda_{irr},T}\left(t\right)}}{A_{tot}^{\lambda_{irr}T}\left(t\right)}\;=A_{X}^{\lambda_{irr},T}\left(t\right)\;P_{0}^{\lambda_{irr}}\;PKF^{\lambda_{irr},T}\left(t\right)$$

The explicit equation of the dimensionless photokinetic factor ($PKF^{\lambda_{irr},T}\left(t\right)$) can be deduced from Equation (4).

The rate constant ($k_{bim}$) of the bimolecular thermal reaction in Equation (3) has a dimension of ${mol}^{-1}\;{dm}^{3}\;or\;M^{-1}$, since the einstein represents a mol of photons. Hence, the left- and the right-hand sides of Equation (3) are expressed in the same units ($M\;s^{-1}=einstein\;{dm}^{-3}s^{-1}$).

The absorbance of the reactant at $\lambda_{irr}$ and time t, $A_{X}^{\lambda_{irr},T}\left(t\right)$, is the product of the decadic absorptivity, ε, the optical path length of the incident irradiation light inside the sample, $l_{irr}$ (in cm), and the concentration, $C_{X}^{\lambda_{irr},T}\left(t\right)$ (in $mol\;{dm}^{-3}$) of X at a given time t (in s), $A_{X}^{\lambda_{irr},T}\left(t\right)=\varepsilon_{X}^{\lambda_{irr}}\;l_{irr}\;C_{X}^{\lambda_{irr},T}\left(t\right)$. The total medium absorbance ($A_{tot}^{\lambda_{irr}}\left(t\right)$) at $\lambda_{irr}$ is expressed for Scheme 1a by a sum,

(5) $$A_{tot}^{\lambda_{irr}T}\left(t\right)=A_{X}^{\lambda_{irr},T}\left(t\right)+A_{X^{\prime }}^{\lambda_{irr},T}\left(t\right)+A_{Y^{\prime }}^{\lambda_{irr},T}\left(t\right)\;$$

The optical path length of observation, $l_{obs}$, related to the monitoring beam of the spectrophotometer measuring the medium absorbances, might be different from that of the irradiation light ($l_{irr}$), and the values of $A^{\lambda_{irr},T}\left(t\right)$ might be inaccessible to direct spectrophotometric measurement. In such a situation, and for the purpose of generalisation of the equations, the measured spectrophotometric value of the absorbance ($A^{\lambda_{obs}}\left(t\right)$ along $l_{obs}$) is used to calculate the absorbance along $l_{irr}$, by the following formula (assuming that the Beer–Lambert law applies in both cases, and both absorbance values fall within the linearity range of the absorbance calibration graphs).

(6) $$A^{\lambda_{irr}}\left(t\right)=A^{\lambda_{obs}}\left(t\right)\;\frac{l_{irr}}{l_{obs}}\;$$

For the reactions considered under polychromatic light, Equation (3) will become an integro-differential equation as it now involves an integration over wavelength (covering the light profile of the lamp used). Such equations cannot be integrated in closed form, as was shown for the equivalent rate laws of unimolecular photo and photothermal reactions (involving a single reactant) [44,59].

### 2.4. The Solvable Rate Equation for the Bimolecular Photoreaction (a)

The rate law given by the latter equation (Equation (3)) is not solvable using the conventional mathematical methods. Several reasons stand behind this fact, with the most relevant being the non-linearity of the differential equation due to the non-linearity of the time-dependent photokinetic factor, $PKF^{\lambda_{irr},T}\left(t\right)$, which depends on the various species concentrations varying during the reaction progress. Analogous situations have been reported to occur for most of the rate equations characterising single, mono-reactant photoreactions under monochromatic, non-isosbestic, and collimated light [44,58]. In all the above cases, the resulting expressions involve integrands that have no known antiderivatives (i.e., the integrand has no know canonical integration form). Consequently, and as a matter of fact, analytically derived integrated rate laws are still lacking in the photochemistry literature for bimolecular photoreactions.

This situation can, however, be alleviated so have integrable rate laws in a few cases. This is achieved by carefully defining the reaction conditions where a closed-form integration becomes possible. These particular cases are discussed below.

**Figure 1:**
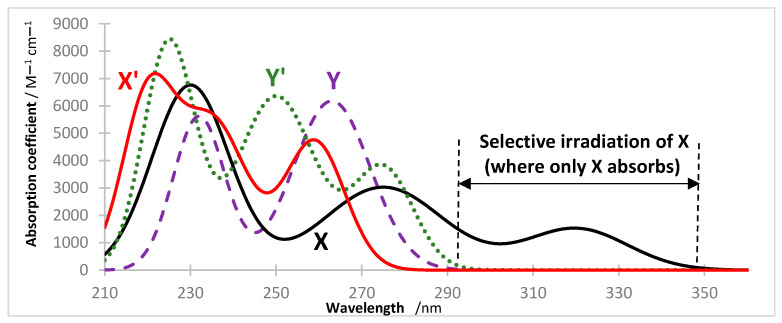
An example of the expected layout of the spectra of the reaction species, showing the region where only reactant X absorbs the monochromatic excitation light (for the acquisition of the species’ individual spectra, see Section 2.7).

Let us first consider Equation (2) for the primary photoprocess, X→Y. The only exception to the above general observation relative to the impossibility of integration of the rate law has been demonstrated for the case where the reactant of the unimolecular reaction, X, is the unique light-absorbing species in the medium at the wavelength of the monochromatic irradiating light (Figure 1). The rate law of the primary photoprocess, considered under non-isosbestic irradiation at $\lambda_{irr}$, and with $\varepsilon_{Y}^{\lambda_{irr}}=0$ (in Equation (2)), accepts a closed-form integration that generates a logarithmic function bearing an exponential in its argument, with a typical $\alpha \;Log(1+\beta \;e^{-\;\gamma \;t})$ formulation (*vide infra* a template in Equation (13)). It represents the first analytically derived integrated rate law equations of the primary photoprocess [57]. This integrated rate law stands for the characteristic formulation of the “Φ*-order kinetics*” [44,58]. It was later proven to fit the kinetic behaviour of any unimolecular photo- or photothermal reaction kinetics [57,58,59], and demonstrated efficiency in the determination of species quantum yields for many molecules, and standardising new and reliable kinactinometers [58,59,60,61,62,63]. The Φ-order kinetics properties have previously been reviewed in detail [44,57,58,59], some of which are described in Section 2.5. 

The validity of the Φ-order equation underlines the necessity for the absorbance of the reactant to fall within the limits of the linearity range (LLR) of the absorbance calibration graph of that species. Otherwise, the coherence and mathematical integrity of the rate equation are lost. The condition on the absorbance of the reactant to fall within the LLR also applies to bimolecular photoreactions. (Beyond these conditions, Equation (2) will have no coherence, i.e., for situations where light collimation is lost, and/or when heterogeneity/deviation from Beer–Lambert linearity occurs).

In order to find the cases where Equation (3) becomes analytically integrable, it is required to implement one more condition in addition to the ones stated above, i.e., (*i*) only X absorbs the incident excitation light in the reactive medium (i.e., $A_{X^{\prime }}^{\lambda_{irr},T}\left(t\right)=A_{Y^{\prime }}^{\lambda_{irr},T}\left(t\right)=0$ in Equation (5)), (*ii*) the irradiation is performed with a monochromatic and collimated light beam at a non-isosbestic wavelength $\lambda_{irr}$, and (*iii*) the absorbance of X used in the experiment must fall within the reactant’s LLR.

Taking conditions (*i*)–(*iii*) into account, the rate of light absorption (Equation (4)) reduces to a much simpler form.

(7) $$P_{a_{X}}^{\lambda_{irr}}\left(t\right)=P_{0}^{\lambda_{irr}}\;\left(1-10^{-A_{X}^{\lambda_{irr},T}\left(t\right)}\right)\;$$

Nonetheless, even when introducing Equation (7) in Equation (3), the latter remains non-integrable. Also, proceeding to a change of variable where the concentration of $X^{\prime }$ is put into the alternative formulation, $C_{X^{\prime }}^{\lambda_{irr},T}\left(t\right)=C_{X^{\prime }}^{\lambda_{irr},T}\left(0\right)-\left(C_{X}^{\lambda_{irr},T}\left(0\right)-C_{X}^{\lambda_{irr},T}\left(t\right)\right)$ (assuming $C_{X^{\prime }}^{\lambda_{irr},T}\left(0\right)>C_{X}^{\lambda_{irr},T}\left(0\right)$), does not solve the problem, even though now only one variable appears in the whole Equation (3). The absence of a known antiderivative for the new Equation (3) implies the need for an additional condition. That is, (*iv*) the concentration of the second reactant ($X^{\prime }$) must be high enough compared to that of X, so that $C_{X^{\prime }}^{\lambda irr,T}\left(t\right)$ is to be considered invariant from its initial value when X is totally depleted. Hence, by considering $C_{X^{\prime }}^{\lambda irr,T}\left(t\right)\gg C_{X}^{\lambda irr,T}\left(0\right)$, the concentration of $X^{\prime }$ must satisfy $C_{X^{\prime }}^{\lambda irr,T}\left(t\right)≅C_{X^{\prime }}^{\lambda irr,T}\left(0\right)$ at any reaction time (it is estimated that the equations stay valid up to a value of $C_{X}^{\lambda irr,T}\left(0\right)={0.10\;C}_{X^{\prime }}^{\lambda irr,T}\left(0\right)$).

Accordingly, Equation (3) takes the following form (where the three terms are multiplied by the factor: $\varepsilon_{X}^{\lambda_{irr}}\;l_{irr}$).

(8) $$\begin{array}{cc} r_{A,X}^{\lambda_{irr},T}\left(t\right)= & r_{X}^{\lambda_{irr},T}\left(t\right)\;\varepsilon_{X}^{\lambda_{irr}}\;l_{irr}=\frac{dA_{X}^{\lambda_{irr},T}\left(t\right)}{dt}=\;\;\;\\ & \;\;-k_{bim}\;C_{X^{\prime }}^{\lambda_{irr},T}\left(0\right)\;P_{0}^{\lambda_{irr}}\;\varepsilon_{X}^{\lambda_{irr}}\;l_{irr}\;\left(1-10^{-A_{X}^{\lambda_{irr},T}\left(t\right)}\right)\; \end{array}$$

The coefficients outside the bracket (in the last term of Equation (8)) are constant with time. This differential equation is non-linear (due to the present of the power number in the bracket), but Equation (8) is, nonetheless, analytically integrable. The reason is the relative simplicity of the formulation of the non-linear term and its dependence on a single concentration, $C_{X}^{\lambda irr,T}\left(t\right)$.

### 2.5. The Integrated Rate Equation for the Bimolecular Photoreaction (a)

The integration procedure of Equation (8), implemented below, mimics the previous method adopted for the solution of Equation (2) [44,57]. Starting by rearranging Equation (8) to separate the variables, as

(9) $$\frac{dA_{X}^{\lambda_{irr},T}\left(t\right)}{1-10^{-A_{X}^{\lambda_{irr},T}\left(t\right)}}=-k_{bim}\;C_{X^{\prime }}^{\lambda_{irr},T}\left(0\right)\;P_{0}^{\lambda_{irr}}\;\varepsilon_{X}^{\lambda_{irr}}\;l_{irr}\;dt\;$$

Then, we proceed to a change of variable on the left-hand side term of Equation (9), where $Z\left(t\right)=10^{A_{X}^{\lambda_{irr},T}\left(t\right)}-1$, and $dZ\left(t\right)=\ln\left(10\right)\;10^{A_{X}^{\lambda_{irr},T}\left(t\right)}\;dA_{X}^{\lambda_{irr},T}\left(t\right)$, as

(10) $$\frac{dA_{X}^{\lambda_{irr},T}\left(t\right)}{1-10^{-A_{X}^{\lambda_{irr},T}\left(t\right)}}=\frac{\ln\left(10\right)\;}{\ln\left(10\right)\;}\;\frac{10^{A_{X}^{\lambda_{irr},T}\left(t\right)}\;dA_{X}^{\lambda_{irr},T}\left(t\right)}{10^{\;A_{X}^{\lambda_{irr},T}\left(t\right)}-1}=\frac{1}{\ln\left(10\right)}\frac{dZ\left(t\right)}{Z(t)}\;$$

which leads to the reformulation of Equation (9) as

(11) $$\frac{dZ\left(t\right)}{Z(t)}=-\ln\left(10\right)\;k_{bim}\;C_{X^{\prime }}^{\lambda_{irr},T}\left(0\right)\;P_{0}^{\lambda_{irr}}\;\varepsilon_{X}^{\lambda_{irr}}\;l_{irr}\;dt\;$$

Then by independently integrating the two terms of Equation (11), and replacing Z(t) by its expression, we obtain

(12) $$ln\frac{Z\left(t\right)}{Z(0)}=ln\frac{10^{\;A_{X}^{\lambda_{irr},T}\left(t\right)}-1}{10^{\;A_{X}^{\lambda_{irr},T}\left(0\right)}-1}=-k_{bim}\;C_{X^{\prime }}^{\lambda_{irr},T}\left(0\right)\;P_{0}^{\lambda_{irr}}\;\varepsilon_{X}^{\lambda_{irr}}\;l_{irr}\;\ln\left(10\right)\;t\;$$

And finally, by extracting the absorbance of X, we derive the following integrated rate law for reaction (*a*), Equation (13). It is written here for the absorbance of X, from which the expression of $C_{X}^{\lambda_{irr},T}\left(t\right)$ can easily be worked out. The occurrence of the decimal logarithm in Equation (13) is consequent to the removal of the power 10 (allowing us to have $A_{X}^{\lambda_{irr},T}\left(t\right)$). The formula for $Y^{\prime }$ is deduced as $C_{Y^{\prime }}^{\lambda_{irr},T}\left(t\right)=C_{X}^{\lambda_{irr},T}\left(0\right)-C_{X}^{\lambda_{irr},T}\left(t\right)$.

(13) $$A_{X}^{\lambda_{irr},T}\left(t\right)=Log\left(1+\left(10^{\;A_{X}^{\lambda_{irr},T}\left(0\right)}-1\right)\;e^{-k_{r}^{bim}\;t}\right)$$

The coefficient of the exponential function ($k_{r}^{bim}$) is

(14) $$k_{r}^{bim}=k_{bim}\;C_{X^{\prime }}^{\lambda_{irr},T}\left(0\right)\;P_{0}^{\lambda_{irr}}\;\varepsilon_{X}^{\lambda_{irr}}\;l_{irr}\;\ln10\;$$

The dimension analysis of $k_{r}^{bim}$ confer a unit $s^{-1}$ (also compatible with the requirement of a dimension 1 for the coefficient of the exponential function). This quantity stands for the overall rate constant of the bimolecular photoreaction considered here under the four ((*i*)–(*iv*)) conditions stated above.

It is obvious that Equation (13) does not correspond to the general reciprocal form of the integrated rate law of a second-order reaction (e.g., $C_{X}\left(t\right)=C_{X}\left(0\right)/\left(1+k\;C_{X}\left(0\right)\;t\right)$). It neither fits the mono-exponential formulation of the pseudo-first-order kinetics (considering that we assumed $C_{X^{\prime }}^{\lambda irr,T}\left(t\right)\gg C_{X}^{\lambda irr,T}\left(0\right)$). Equation (13) is, however, a typical integrated rate law of Φ-order kinetics.

Equation (13) hence follows the characteristic behaviour of Φ-order reactions [44,57,58]. Its rate constant (Equation (14)) is independent of time and the initial concentration of reactant X, but dependent on the remaining reaction parameters (including the irradiated area and volume of the sample, which are implicit in the formula of the incident light $P_{0}^{\lambda_{irr}}$ [44,58]). However, the overall bimolecular photoreaction, $k_{r}^{bim}$ is also dependent on the medium temperature via the involvement of the rate constant $k_{bim}$ of the bimolecular thermal reaction ($k_{bim}$ is a temperature-dependent quantity).

**Figure 2:**
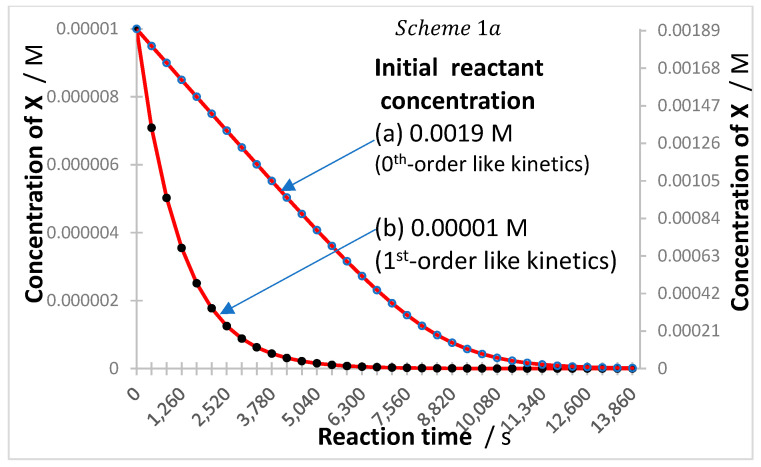
Dependence of the shape of the reactant trace on its initial concentration. A first-order-type curve is obtained when $C_{X}^{\lambda irr,T}\left(0\right)$ is low ($10^{-5}\;M$), but rather linear, of zero-order type, for high values of $C_{X}^{\lambda irr,T}\left(0\right)=1.9\times 10^{-3}\;M$. The dots and circles correspond to the values of the numerical integrated of the rate law (Equation (11)), whereas the fitting lines correspond to Equation (14). The reaction features are $k_{bim}=0.4\;M^{-1}$, $l_{irr}=1\;cm$, $C_{X^{\prime }}^{\lambda irr,T}\left(0\right)=1.7\times 10^{-2}\;M$, $P_{0}^{\lambda_{irr}}=3.33\times 10^{-4}\;einstein\;{dm}^{-3}s^{-1}$, $\varepsilon_{X}^{\lambda_{irr}}=1593\;M^{-1}\;{cm}^{-1}$, and the overall rate constant (Equation (15)) is $k_{r}^{bim}=8.31\times 10^{-4}\;s^{-1}$.

Also, according to the properties of Φ-order kinetics, in the limit case where $C_{X}^{\lambda irr,T}\left(0\right)$ is either relatively small or relatively high (but with $C_{X}^{\lambda irr,T}\left(0\right)\ll C_{X^{\prime }}^{\lambda irr,T}\left(0\right)$, and where the four conditions (*i*)–(*iv*) apply), the Φ-order equation tends to behave, respectively, either as a mono-exponential function (first-order kinetics) or as a linear trend (zeroth-order kinetics) [44,58]. Figure 2 depicts an example of traces corresponding to these limiting cases.

Accordingly, there is a true change of the bimolecular photoreaction order, taking one of three options depending on the initial concentration values of X (first-, zeroth-, or Φ-order). This represents the first photokinetic-based explanation ever provided in the literature for the kinetic order changes of bimolecular photoreactions.

Even if this might explain the usage of the pseudo-first order kinetics for such a reaction in the literature, it is, however, important to underline that the rate constant defined from the pseudo-first order (mono-exponential) formula does not correspond to or equate that ($k_{r}^{bim}$, Equation (14)) obtained from the application of Equation (13). In general, the first-order rate constant (obtained directly from fitting the data of $C_{X}^{\lambda_{irr},T}\left(t\right)$ with a mono-exponential function) has the expression $k_{r}=k_{bim}\;C_{X^{\prime }}^{\lambda_{irr},T}\left(0\right)\;P_{0}^{\lambda_{irr}}\;\varepsilon_{X}^{\lambda_{irr}}\;l_{irr}$. It is obvious that $k_{r}$ does not correspond exactly to the rate constant $k_{bim}$ of the bimolecular thermal reaction. It includes both the absorptivity of the reactant and three other experimental features ($C_{X^{\prime }}^{\lambda_{irr},T}\left(0\right)\;P_{0}^{\lambda_{irr}}\;l_{irr}$). However, $k_{r}$ has the same dependence on temperature as $k_{bim}$.

Also, theoretically, the reciprocal form of the second-order integrated rate law cannot, mathematically, be derived from Equation (13) either for the general bimolecular photoreaction or for any specific cases involving particular experimental conditions.

### 2.6. The Bimolecular Photoreaction (b) Involving a Concurrent Photolysis of the Reactant

The rate equation describing the reactant evolution, for the bimolecular reaction depicted in Scheme 1b, considered here under the same four conditions (*i*)–(*iv*) stated above (Section 2.4), is a combination of Equations (2) and (8), as

(15) $$\begin{array}{ll} r_{X}^{\lambda_{irr},T}\left(t\right)\;\varepsilon_{X}^{\lambda_{irr}}\; & l_{irr}=\frac{dA_{X}^{\lambda_{irr},T}\left(t\right)}{dt}\\ & \;=-\left(k_{bim}\;C_{X^{\prime }}^{\lambda_{irr},T}\left(0\right)+\Phi_{X\;\to \;Y}^{\lambda_{irr}}\right)\;P_{0}^{\lambda_{irr}}\;\varepsilon_{X}^{\lambda_{irr}}\;l_{irr}\;\left(1-10^{-A_{X}^{\lambda_{irr},T}\left(t\right)}\right)\; \end{array}$$

The solution of Equation (15) for the reactant is achieved by the method used to solve Equation (8). The derived integrated rate law has the same Φ-order expression depicted by Equation (13), except that the rate constant is now inclusive of the two processes (Equation (16)).

(16) $$k_{r,X}^{bim}=\left(k_{bim}\;C_{X^{\prime }}^{\lambda_{irr},T}\left(0\right)+\Phi_{X\;\to \;Y}^{\lambda_{irr}}\right)\;P_{0}^{\lambda_{irr}}\;\varepsilon_{X}^{\lambda_{irr}}\;l_{irr}\ln10\;$$

The description of the kinetic traces belonging to the two products (Y and Y′, Scheme 1b) of the reaction is worked out as follows.

The rate law of Y, is a part of Equation (15), i.e., without the bimolecular rate constant.

(17) $$r_{Y}^{\lambda_{irr},T}\left(t\right)=\frac{dC_{Y}^{\lambda_{irr},T}\left(t\right)}{dt}=\Phi_{X\;\to \;Y}^{\lambda_{irr}}\;P_{0}^{\lambda_{irr}}\left(1-10^{-A_{X}^{\lambda_{irr},T}\left(t\right)}\right)\;$$

The solving procedure of this equation starts by rearranging Equation (17), rewriting the term $1-10^{-\;A_{X}^{\lambda_{irr},T}\left(t\right)}$, then introducing the formula of $A_{X}^{\lambda_{irr},T}\left(t\right)$ (Equation (13)) in the resulting equation, to make its right-hand side explicitly dependent on time only. We further proceed to a change of variable $(W\left(t\right)=1+\left(10^{\;A_{X}^{\lambda_{irr},T}\left(0\right)}-1\right)\;e^{-\;k_{r}^{bim}\;t}$, with $dW\left(t\right)=-k_{r}^{bim}\;\left(10^{\;A_{X}^{\lambda_{irr},T}\left(0\right)}-1\right)\;e^{-k_{r}^{bim}\;t})$. Accordingly, the left-hand side term of the new equation transforms as follows.

(18) $$\begin{array}{l} \left(1-10^{-A_{X}^{\lambda_{irr},T}\left(t\right)}\right)dt=\frac{10^{A_{X}^{\lambda_{irr},T}\left(t\right)}-1}{10^{A_{X}^{\lambda_{irr},T}\left(t\right)}}dt=\\ \;\;\;\frac{-1}{k_{r}^{bim}}\;\frac{-k_{r}^{bim}\;\left(10^{\;A_{X}^{\lambda_{irr},T}\left(0\right)}-1\right)\;e^{-k_{r}^{bim}\;t}}{1+\left(10^{\;A_{X}^{\lambda_{irr},T}\left(0\right)}-1\right)\;e^{-k_{r}^{bim}\;t}}dt=-\frac{1}{k_{r}^{bim}}\;\frac{dW(t)}{W(t)}\; \end{array}$$

So that Equation (17) becomes

(19) $$dC_{Y}^{\lambda_{irr},T}\left(t\right)=\Phi_{X\;\to \;Y}^{\lambda_{irr}}\;P_{0}^{\lambda_{irr}}\frac{-1}{k_{r}^{bim}}\;\frac{dW(t)}{W(t)}\;$$

Which is readily integrated into

(20) $$C_{Y}^{\lambda_{irr},T}\left(t\right)=\Phi_{X\;\to \;Y}^{\lambda_{irr}}\;P_{0}^{\lambda_{irr}}\;\frac{-1}{k_{r}^{bim}}\;ln\left(\frac{W(t)}{W(0)}\right)\;$$

Or by replacing W(t) in Equation (20) with its expression, and rearranging the resulting equation, we finally have

(21) $$\begin{array}{ll} C_{Y}^{\lambda_{irr},T}\left(t\right)= & -\frac{1}{\varepsilon_{X}^{\lambda_{irr}}\;l_{irr}}\;\frac{\Phi_{X\;\to \;Y}^{\lambda_{irr}}}{k_{bim}\;C_{X^{\prime }}^{\lambda_{irr},T}\left(0\right)+\Phi_{X\;\to \;Y}^{\lambda_{irr}}}\times \\ & \;\;log\left(10^{\;{-A}_{X}^{\lambda_{irr},T}\left(0\right)}+\left(1-10^{-A_{X}^{\lambda_{irr},T}\left(0\right)}\right)\;e^{-k_{r}^{bim}\;t}\right)\; \end{array}$$

The limit value of $C_{Y}^{\lambda_{irr},T}\left(t\right)$ at the initial time, is $C_{Y}^{\lambda_{irr},T}\left(0\right)=0$, and that for a long reaction time (t=∞),

(22) $$C_{Y}^{\lambda_{irr},T}\left(\infty \right)=C_{X}^{\lambda_{irr},T}\left(0\right)\frac{\;\Phi_{X\;\to \;Y}^{\lambda_{irr}}}{k_{bim}\;C_{X^{\prime }}^{\lambda_{irr},T}\left(0\right)+\Phi_{X\;\to \;Y}^{\lambda_{irr}}}\;$$

Let us now consider the rate law of the second product $Y^{\prime }$ of the reaction,

(23) $$r_{Y^{\prime }}^{\lambda_{irr},T}\left(t\right)=\frac{dC_{Y^{\prime }}^{\lambda_{irr},T}\left(t\right)}{dt}=k_{bim}\;C_{X^{\prime }}^{\lambda_{irr},T}\left(0\right)\;P_{0}^{\lambda_{irr}}\;\left(1-10^{-A_{X}^{\lambda_{irr},T}\left(t\right)}\right)\;$$

We employ here the same procedure that led to Equation (21) to obtain the integrated rate law for $C_{Y^{\prime }}^{\lambda_{irr},T}\left(t\right)$, as

(24) $$\begin{array}{ll} C_{Y^{\prime }}^{\lambda_{irr},T}\left(t\right)= & -\frac{1}{\varepsilon_{X}^{\lambda_{irr}}\;l_{irr}}\;\frac{k_{bim}\;C_{X^{\prime }}^{\lambda_{irr},T}\left(0\right)}{k_{bim}\;C_{X^{\prime }}^{\lambda_{irr},T}\left(0\right)+\Phi_{X\;\to \;Y}^{\lambda_{irr}}}\times \\ & \;log\left(10^{\;{-A}_{X}^{\lambda_{irr},T}\left(0\right)}+\left(1-10^{-A_{X}^{\lambda_{irr},T}\left(0\right)}\right)\;e^{-k_{r}^{bim}\;t}\right)\; \end{array}$$

With at t=0, $C_{Y^{\prime }}^{\lambda_{irr},T}\left(0\right)=0$, and at t=∞,

(25) $$C_{Y^{\prime }}^{\lambda_{irr},T}\left(\infty \right)=C_{X}^{\lambda_{irr},T}\left(0\right)\;\frac{k_{bim}\;C_{X^{\prime }}^{\lambda_{irr},T}\left(0\right)}{k_{bim}\;C_{X^{\prime }}^{\lambda_{irr},T}\left(0\right)+\Phi_{X\;\to \;Y}^{\lambda_{irr}}}\;$$

The mass balance is expressed as

(26) $$C_{X}^{\lambda_{irr},T}\left(0\right)=C_{X}^{\lambda_{irr},T}\left(t\right)+C_{Y}^{\lambda_{irr},T}\left(t\right)+C_{Y^{\prime }}^{\lambda_{irr},T}\left(t\right)=C_{Y}^{\lambda_{irr},T}\left(\infty \right)+C_{Y^{\prime }}^{\lambda_{irr},T}\left(\infty \right)\;$$

**Figure 3:**
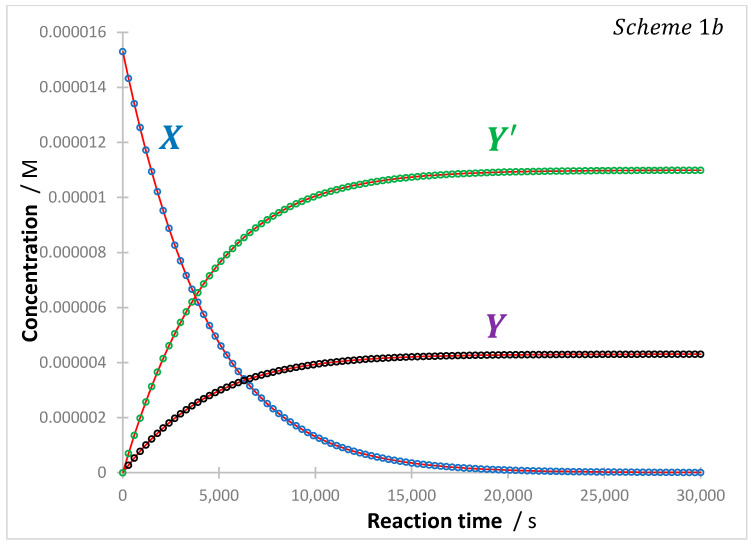
Kinetic traces of the three ($X,\;Y\;and\;Y^{\prime }$) species of reaction (*b*) as depicted in Scheme 1. An excellent fitting of the numerically calculated data (circles), performed on the rate equations Equations (16), (18) and (24), by the analytically derived concentrations’ expressions Equations (13), (21), and (24), respectively, for $X,\;Y\;and\;Y^{\prime }$ (red lines). The reaction properties are: $C_{X}^{\lambda irr,T}\left(0\right)=1.53\times 10^{-5}\;M$, $C_{X^{\prime }}^{\lambda irr,T}\left(0\right)=9.82\times 10^{-3}\;M$, $l_{irr}=1.16\;cm$, $\varepsilon_{X}^{\lambda_{irr}}=10,202\;M^{-1}\;{cm}^{-1}$, $P_{0}^{\lambda_{irr}}=1.37\times 10^{-6}\;einstein\;{dm}^{-3}s^{-1}$, $k_{bim}=0.52\;M^{-1}$, $\Phi_{X\;\to \;Y}^{\lambda_{irr}}=2\times 10^{-3}$, and the overall rate constant (Equation (14)) is $k_{r}^{bim}=2.65\times 10^{-4}\;s^{-1}$.

Accordingly, the three species of reaction (*b*) obey Φ-order kinetics (as did X and $Y^{\prime }$ for reaction (*a*)). Figure 3 provides an example of the photokinetic traces of this reaction.

### 2.7. Generalisation and Quantification

**Scheme 2:**
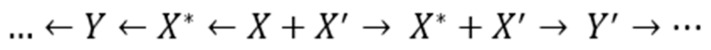
Extended mechanism of the bimolecular reaction given in Scheme 1b.

As long as the conditions (*i*)–(*iv*) stated in Section 2.4 are satisfied (e.g., $X^{\prime }$ and all the products are transparent to the incident light at the irradiation wavelength, $\lambda_{irr}$), and the reactant direct photolysis starts with a unimolecular $X\overset{hv}{\to }Y$ reaction, then irrespective of later photo- or photothermal mechanisms undergone by the products Y and Y′ (Scheme 2), the kinetics behaviour of X will still have a Φ-order character (i.e., be described by Equation (13)).

In terms of quantification, the quantum yield of the direct photolysis ($\Phi_{X\;\to \;Y}^{\lambda_{irr}}$) can be determined by a separate experiment where X is irradiated alone in the same conditions of irradiation of the bimolecular photoreaction. The trace of X can be acquired spectrophotometrically as at $\lambda_{irr}$ where only X absorbs, but can also be obtained by employing, for instance, a separation method such as HPLC. The trace is then fitted with Equation (13), which has $k_{r}^{bim}$ as the unique fitting parameter. The kinetics of unimolecular photoreaction have been previously fully elucidated [44,58]. In this method, the absolute value of the quantum yield is worked out from the expression of the rate constant $k_{X\;\to \;Y}^{\lambda_{irr}}$ as Equation (16) where $k_{bim}=0$ (since the other coefficients in that equation are experimentally accessible). Otherwise, the value of the initial rate of the reaction ($Fit:r_{0,X}^{\lambda_{irr},T}$) can be calculated from the fitting parameters, and thereafter, used to determine the true value of $\Phi_{X\;\to \;Y}^{\lambda_{irr}}$, as

(27) $$\Phi_{X\;\to \;Y}^{\lambda_{irr}}=-\frac{Fit:r_{0,X}^{\lambda_{irr},T}}{\;P_{0}^{\lambda_{irr}}\;\left(1-10^{-A_{X}^{\lambda_{irr},T}\left(0\right)}\right)}\;$$

The rate constant of the thermal bimolecular reaction ($k_{bim}$) can then be extracted from the rate constant of the overall bimolecular reaction ($k_{r}^{bim}$, Equation (16)), whose value is worked out from Equation (13) (by fitting that equation to the trace of X obtained from the bimolecular photoreaction).

The absorptivity spectra of the reactants X and $X^{\prime }$ ($\varepsilon_{X}^{\lambda_{irr}}$ and $\varepsilon_{X^{\prime }}^{\lambda_{irr}}$) can be worked out from their individual electronic absorption spectra obtained from their individual solutions and their calibration graphs. The spectrum of the unimolecular photoreaction’s product, Y, is worked out from the absorption spectrum of the solution obtained by a complete photolysis of X (present alone in the reactor). The absorptivity spectrum of $Y^{\prime }$ might be constructed from the final absorption spectrum for the reaction after a long time (since we know $C_{Y^{\prime }}^{\lambda_{irr},T}\left(\infty \right)$ from Equation (25)). In this case, the total absorption spectrum recorded on the reactive medium at t = ∞ corresponds to the absorptions of $X^{\prime }$, $Y^{\prime }$, and Y (if the latter is present in the medium), hence, $A_{Y^{\prime }}^{\lambda_{irr},T}\left(\infty \right)=A_{tot}^{\lambda_{irr}T}\left(\infty \right)-A_{X^{\prime }}^{\lambda_{irr},T}\left(0\right)-A_{Y}^{\lambda_{irr},T}\left(\infty \right)$.

### 2.8. The Bimolecular Photoreaction (c)

The system of differential equations describing the species of reaction (*c*), considered under the conditions ((*i*)–(*iv*)), is

(28) $$r_{X}^{\lambda_{irr},T}\left(t\right)=-r_{Y^{\prime \prime }}^{\lambda_{irr},T}\left(t\right)=-\Phi_{X\;\to \;Y^{\prime },Y^{\prime \prime }}^{\lambda_{irr}}\;P_{0}^{\lambda_{irr}}\;\left(1-10^{-A_{X}^{\lambda_{irr},T}\left(t\right)}\right)\;$$

(29) $$r_{Y^{\prime }}^{\lambda_{irr},T}\left(t\right)=-k_{bim}\;C_{X^{\prime }}^{\lambda_{irr},T}\left(0\right)\;C_{Y^{\prime }}^{\lambda_{irr},T}\left(t\right)+\Phi_{X\;\to \;Y}^{\lambda_{irr}}\;P_{0}^{\lambda_{irr}}\;\left(1-10^{-A_{X}^{\lambda_{irr},T}\left(t\right)}\right)\;$$

(30) $$r_{Y^{\prime \prime \prime }}^{\lambda_{irr},T}\left(t\right)=k_{bim}\;C_{X^{\prime }}^{\lambda_{irr},T}\left(0\right)\;C_{Y^{\prime }}^{\lambda_{irr},T}\left(t\right)\;$$

The rate of the first reactant ($r_{X}^{\lambda_{irr},T}\left(t\right)$) is solvable by the same method used in Section 2.5. The variation of the concentration of X, when considered alone, follows Φ-order kinetics according to the formulation of Equation (13), with a rate constant $k_{X\;\to \;Y^{\prime },Y^{\prime \prime }}^{\lambda_{irr}}$ of the type given by Equation (16), where $k_{bim}=0$. The same kinetic behaviour should characterise the product $Y^{\prime \prime }$ of reaction (*c*), with $C_{Y^{\prime \prime }}^{\lambda_{irr},T}\left(t\right)=C_{X}^{\lambda_{irr},T}\left(0\right)-C_{X}^{\lambda_{irr},T}\left(t\right)$.

However, the behaviours of the two other species ($Y^{\prime }$ and $Y^{\prime \prime \prime }$, Scheme 1c) in this reaction remain with no known mathematical template, because analytical integrations of Equations (29) and (30) are unachievable. The reasons are the impossibility of proceeding with a separation of variables, for instance, in the rate $r_{Y^{\prime }}^{\lambda_{irr},T}\left(t\right)$, and to express the $C_{Y^{\prime }}^{\lambda_{irr},T}\left(t\right)$ as a function of $C_{X}^{\lambda_{irr},T}\left(t\right)$ (because the explicit expression of the latter is not known and $C_{Y^{\prime }}^{\lambda_{irr},T}\left(t\right)\neq C_{X}^{\lambda_{irr},T}\left(0\right)-C_{X}^{\lambda_{irr},T}\left(t\right)$).

**Figure 4:**
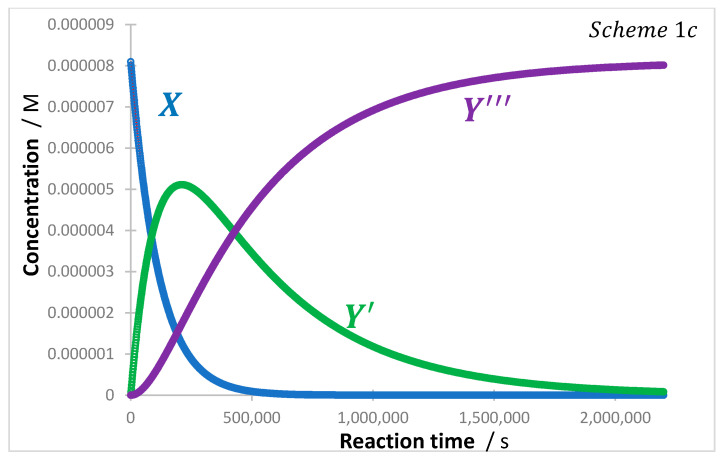
Numerically calculated traces of $X,\;Y^{\prime }\;and\;Y^{\prime \prime }$ species of reaction (*c*) (circles). The kinetic trace of X is well fitted by Equation (13) (red line). The reaction conditions are given in the text.

The numerical integration of the system of differential equations (Equations (28)–(30)) can be performed to produce the traces of the individual reaction species. An illustration example is shown in Figure 4 for the following reaction features $C_{X}^{\lambda irr,T}\left(0\right)=8.1\times 10^{-6}\;M$, $C_{X^{\prime }}^{\lambda irr,T}\left(0\right)=1.3\times 10^{-4}\;M$, $l_{irr}=0.86\;cm$, $\varepsilon_{X}^{\lambda_{irr}}=7211\;M^{-1}\;{cm}^{-1}$, $P_{0}^{\lambda_{irr}}=1.2\times 10^{-6}\;einstein\;{dm}^{-3}s^{-1}$, $\Phi_{X\;\to \;Y^{\prime },Y^{\prime \prime }}^{\lambda_{irr}}=5.3\times 10^{-4}$, $k_{bim}=1.7\times 10^{-2}\;M^{-1}$.

The initial rates of the formation of X and $Y^{\prime }$ are equal. The occurrence of two kinetic regimes is evidenced in both the trace of $Y^{\prime }$ (formation from X and depletion to $Y^{\prime \prime \prime }$), and the sigmoid-like shape of $Y^{\prime \prime \prime }$ kinetic trace (Figure 4). Similar behaviours of the latter species have been observed for several photocatalytic processes [23,33,47,50].

Since the integrated rate laws of $Y^{\prime }$ and $Y^{\prime \prime \prime }$ are not accessible, it becomes evident that the value of the rate constant, $k_{bim}$, of the bimolecular thermal reaction can be obtained by numerical integration. This is effectively possible if the absolute value of the quantum yield of the first reactant ($\Phi_{X\;\to \;Y^{\prime },Y^{\prime \prime }}^{\lambda_{irr}}$) is known (determined from the concentration trace of that species as laid out in Section 2.7). In such a case, the numerical calculation can be optimised for $k_{bim}$ as the only unknown of the fitting process. This alternative is viable since many commercially available software packages are capable of performing such a numerical integration.

The above formulae and/or procedures can be found very useful for any reaction whose reactant X absorbs in a separate region of the electromagnetic spectrum to that of the rest of the species present in the reactive medium (Figure 1), such as in the case of many addition reactions where X absorbs in a bathochromic region (e.g., >290 nm) to that of X′ and Y′ absorption (e.g., <280 nm) [64] (for instance, when X is characterised by an extended double bond system whereas the other species involve single or no double bonds); or those of visible light photocatalysis [18,28,65,66,67,68,69,70,71,72,73,74,75], with the photocatalyst absorbs in the visible range of the electromagnetic spectrum while the rest of the species of the bimolecular reaction mainly absorb in the UV.

### 2.9. The Last Bimolecular Photoreactions (d)

The rate law of reaction (*d*), Equation (31), is equally not analytically solvable. Proceeding to the separation of the variable in Equation (31) yields an expression that has no known antiderivative [44]. Of course, the explicit formula of the reactant concentration as a function of time is not available (in fact, $C_{X}^{\lambda_{irr},T}\left(t\right)$ is expected to be derived from solving Equation (31)).

(31) $$\begin{array}{ll} r_{X}^{\lambda_{irr},T}\left(t\right) & =-r_{Y}^{\lambda_{irr},T}\left(t\right)=-k_{bim}\;P_{a_{X}}^{\lambda_{irr}}\left(t\right)\;C_{X}^{\lambda_{irr},T}\left(t\right)=\\ & \;-k_{bim}\;P_{0}^{\lambda_{irr}}\;\left(1-10^{-A_{X}^{\lambda_{irr},T}\left(t\right)}\right)\;C_{X}^{\lambda_{irr},T}\left(t\right)\; \end{array}$$

**Figure 5:**
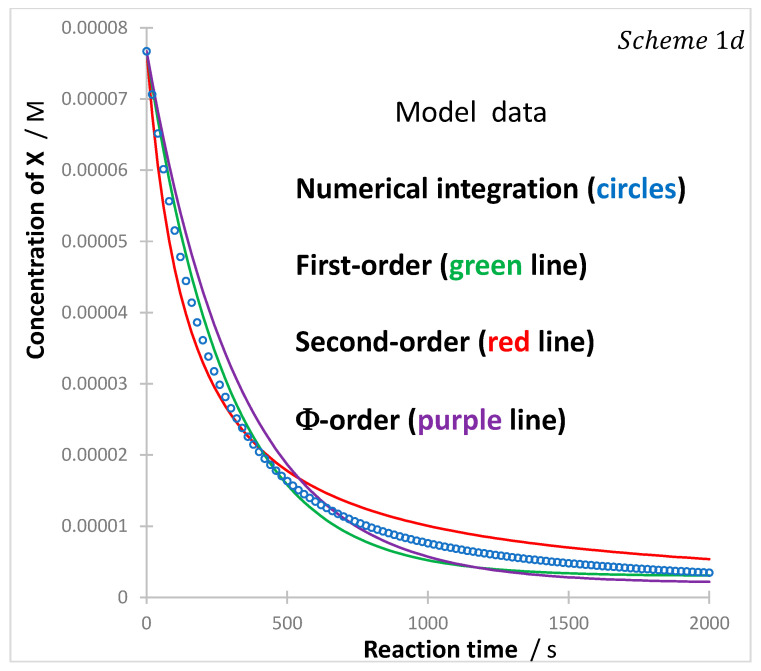
Inadequate fitting of the numerically calculated traces of X (reaction (*d*)) (circles) by the equations of commonly used kinetic model (lines, mono-exponential (green), reciprocal (red), and log-exp as Equation (13) (purple)). The reaction features include $C_{X}^{\lambda irr,T}\left(0\right)=6.67\times 10^{-5}\;M$, $l_{irr}=1.56\;cm$, $\varepsilon_{X}^{\lambda_{irr}}=11,086\;M^{-1}\;{cm}^{-1}$, $P_{0}^{\lambda_{irr}}=9.05\times 10^{-3}\;einstein\;{dm}^{-3}s^{-1}$, $k_{bim}=0.48\;M^{-1}$.

The concentration trace of the reactant, generated numerically, does not seem to be fitted by the classical mono-exponential (first-order kinetics), the reciprocal (second-order kinetics), or the Φ-order kinetic models (Figure 5). Such evidence confirms the approximate character of such models when applied to describe the kinetics of this bimolecular photoreaction (*c*).

Here as well, numerical integration is appropriate for the determination of $k_{bim}$ value (since the latter can be used as an adjustment variable to fit the experimental trace by the numerical integration data).

Incidentally, we notice that for relatively low initial concentrations of X, the coefficient $10^{-\;A_{X}^{\lambda_{irr},T}\left(t\right)}$ in Equation (31) becomes negligible and therefore the reaction will obey first-order kinetics. A situation that justifies the use of first-order kinetics for such reactions, but also clearly indicates a genuine change of the reaction order (since the true order of this reaction would most likely be different from a first-order).

### 2.10. Quantum Yield or Photonic Yield

The quantum yield is specifically defined for primary photochemical processes, i.e., *any elementary chemical process undergone by an electronically excited molecular entity and yielding a primary photoproduct* [46]. In this sense, the quantum yield is not a suitable quantification parameter for bimolecular photoreactions because the reactions’ products are not primary (the absorption of light of one species is coupled with a subsequent thermal reaction, occurring with another species, in order to generate the product). Incidentally, the evaluation of the quantum yield is only to be envisaged for reactions subjected to strictly monochromatic irradiation and must not, at all, be considered whenever the incident light is polychromatic [44,46]. Accordingly, the quantum yield is inadequate to quantify the overall performance of bimolecular photoreactions.

The photonic yield (PY) is the appropriate parameter for the assessment of bimolecular photoreactions in general. The photonic yield, as well as the reactivity of a bimolecular photoreaction, are relative quantities that depend on the reaction conditions and features (including temperature, irradiated volume, and reaction time) [44,59]. Its determined value is specific to those features and conditions, and hence its full definition requires including information about all experimental conditions in which the reaction at hand has been performed.

The dimensionless photonic yield (Equation (32)) is defined as the ratio of the reactive species (e.g., the concentration of X) depleted during reaction time t, and the amount of light received by the reactor in the same interval of time (i.e., received before absorption).

(32) $${PY}_{X}^{\lambda_{irr},T}=\frac{C_{X}^{\lambda_{irr},T}\left(0\right)-C_{X}^{\lambda_{irr},T}\left(t\right)}{\;P_{0}^{\lambda_{irr}}\;t}\;$$

Similar formulae can be written for the second reactant $X^{\prime }$ and the product (e.g., $Y^{\prime }$), by replacing, in Equation (32), X by $X^{\prime }$ or by $Y^{\prime }$ respectively, and introducing a minus sign in the right-hand side of the PY equations of the products, e.g., $-\left(C_{Y^{\prime }}^{\lambda_{irr},T}\left(0\right)-C_{Y^{\prime }}^{\lambda_{irr},T}\left(t\right)\right)$. Notice that while for the reaction depicted in Scheme 1a, the photonic yields of the three species should be equal, i.e., ${PY}_{X}^{\lambda_{irr},T}={PY}_{X^{\prime }}^{\lambda_{irr},T}={PY}_{Y^{\prime }}^{\lambda_{irr},T}$, it must, however, be different for the various species of each of the other bimolecular photoreactions listed in Scheme 1.

As such, the PY formula (Equation (32)) does not relate to the amount of light effectively absorbed but rather to the amount of light ($P_{0}^{\lambda_{irr}}\;t$) received by the reactor (before absorption). In principle, this somewhat contravenes the first law of photochemistry stating that only absorbing species can react. The above PY formula was interpreted as a light-energy efficiency relationship whereby the outcome of the reaction is considered relative to the total energy (or number of photons) reaching the reactor [44,46,59].

## 3. Conclusions

The thermal kinetic models are, in principle, unsuitable for photoreactions. The reason is the significant differences occurring in the mathematical descriptions of the two reactive systems. The rate laws of the latter must explicitly involve both the intensity of the radiation impinging on the sample and its absorption by the reactant and other species present in the medium. Also, the rate laws of photoreactions must only concern the fractions of the first reactant molecules (e.g., X) that can photoreact, i.e., those being in their excited state ($X^{*}$).

In this context, the rate laws of the bimolecular photoreactions, presented in the present paper, encompass the above considerations in that they include explicit coefficients of both the radiation intensity and the fraction of the absorbed light.

The rate laws of photoreactions are, in general, not analytically integrable. A hurdle that is mainly due to the presence, in these rate laws, of the non-linear photokinetic factor expressed as a function of the reaction’s species absorbances. This mostly leads to unsolvable differential equations and/or to the occurrence of integrands whose canonical forms are unknown.

In the case of bimolecular photoreactions, it is, however, possible to analytically integrate some rate laws when considered under specific conditions. Among the four reactions depicted in Scheme 1, the closed-form integration can be achieved for two, if the irradiation is performed with a monochromatic/collimated light, only the first reactant (X) absorbs the incident/excitation light, the concentration of the second reactant (X′) is high, and the concentration of the first reactant falls within the limits of the linearity range of the absorbance calibration graph of that species.

The integrated rate laws prove that reactions (*a*), (*b*), and the first unimolecular photoprocess of reaction (*c*), as depicted in Scheme 1, all obeyed Φ-order kinetics. Even though the conditions allowing such analytical integration are very limiting in practice, they nonetheless allow us to establish, for the first time in the literature, the true kinetic order of these reactions.

The quantification of the unknowns of reactions (*1a*) and (*1b*) is facilitated by the integrated rate law equations (Section 2.5). Also, for the first process in reaction (*1c*), the quantum yield of the primary photoprocess is readily calculated from the rate constant formula (e.g., Equation (16) where $k_{bim}=0$). However, for the unsolvable reaction cases (reactions (*c*) and (*d*)), the photonic yield remains the only overall appropriate quantification means (e.g., Equation (25)). The determination of the thermal rate constants of the non-integrable rate laws corresponding to the bimolecular photoreactions (*c*) and (*d*) can be obtained by employing efficient numerical integration methods such as the fourth-order Runge–Kutta method. These procedures can be applied to experimental data of the bimolecular systems satisfying conditions (*i*)–(*iv*) (and Figure 1).

The findings of the present work provide both a new perspective on the kinetic evaluation and a contribution to the standardisation of the photokinetics of bimolecular reactions.

Further photokinetic analysis of the majority of bimolecular reactions, whose rate laws are not solvable by closed-form integration, would preferably be carried out by the development of predictive modelling strategies offering reliable descriptive and quantification tools.

## Data Availability

Data are contained within the article.
